# The complete chloroplast genome sequence of the medicinal plant *Salvia yunnanensis* C. H. Wright. (Lamiaceae)

**DOI:** 10.1080/23802359.2019.1677523

**Published:** 2019-10-16

**Authors:** Aien Tao, Feiya Zhao, Jinfu Qian

**Affiliations:** School of Medicine, Tourism and Culture College, Yunnan University, Lijiang, Yunnan, China

**Keywords:** *Salvia yunnanensis*, chloroplast, Illumina sequencing, phylogeny

## Abstract

*Salvia yunnanensis* is a medicinal plant commonly used in the southwest of China. In this study, we sequenced the complete chloroplast (cp) genome sequence of *S. yunnanensis* to investigate its phylogenetic relationship in the family Lamiaceae. The total length of the cp genome was 151,338 bp, with 38.0% overall GC content and exhibited typical quadripartite structure, a pair of IRs (inverted repeats) of 25,578 bp each were separated by a small single-copy (SSC) region of 17,564 bp and a large single-copy (LSC) region of 82,618 bp. The cp genome contained 114 genes, including 80 protein coding genes, 30 tRNA genes, and 4 rRNA genes. The phylogenetic analysis indicated *S. yunnanensis* was closely related to *S. miltiorrhiza*, which afforded a scientific evidence that *S. yunnanensis* could be used as original species of Radix et Rhizoma Saliviae Miltiorrhizae (Danshen).

*Salvia* L. is a great genus of the Lamiaceae family, which includes 900 species in the world (Topçu 2006). Most of them are widespread in temperate regions and tropical mountains including Central and South America, Central Asia/Mediterranean and Eastern Asia (Li et al. 2013). There are 84 species in China (Li and Ian 1994) and the majority of them are distributed in southwest China, notably in the Hengduan Mountain region. In some areas of southwest China, *Salvia yunnanensis* is a local medicine commonly used as the surrogate of the traditional Chinese medicine Radix et Rhizoma Saliviae Miltiorrhizae (Danshen, *Salvia miltiorrhiza*) for the treatment of various cardiovascular diseases (Jiangsu New Medical College 1977). However, up to now, for such a medicinal plant, studies have focussed on describing its chemical compositions (Xu et al. 2006; Xiang et al. 2013; Xia et al. 2015) and DNA barcoding analysis (Wang and Wang 2005; Wang et al. 2007), with little involvement in its genomes. Here, we report the chloroplast (cp) genome sequence of *S. yunnanensis* and find its internal relationships within the family Lamiaceae.

Fresh and clean leaf materials of *S. yunnanensis* were collected from Dali county, Yunnan, China (N25.62°, E100.48°), and the plant materials and a voucher specimen (No. LYTE01) were deposited at Tourism and Culture College of Yunnan University (Lijiang). Total genomic DNA was extracted using the improved CTAB method (Doyle 1987; Yang et al. 2014), and sequenced with Illumina Hiseq 2500 (Novogene, Tianjing, China) platform with pair-end (2 × 300 bp) library. About 5.19 Gb of raw reads with 17,290,260 paired-end reads were obtained from high-throughput sequencing. The raw data was filtered using Trimmomatic v.0.32 with default settings (Bolger et al. 2014). Then paired-end reads of clean data were assembled into circular contigs using GetOrganelle.py (Jin et al. 2018) with *Salvia miltiorrhiza* (No. NC_020431) as reference. Finally, the plastome was annotated by the Plastid Genome Annotator (PGA) (Qu et al. 2019) with manual adjustment using Geneious v. 7.1.3 (Kearse et al. 2012).

The circular genome map was generated with OGDRAW v.1.3.1 (Greiner et al. 2019). Then the annotated cp genome was submitted to the GenBank under the accession number MN341012. The total length of the cp genome was 151,338 bp, with 38.0% overall GC content. With typical quadripartite structure, a pair of IRs (inverted repeats) of 25,578 bp was separated by a small single-copy (SSC) region of 17,564 bp and a large single-copy (LSC) region of 82,618 bp. The cp genome contained 114 genes, including 80 protein-coding genes, 30 tRNA genes, and 4 rRNA genes. Of these, 20 genes were duplicated in the inverted repeat regions, 11 genes, and 6 tRNA genes contain one intron, while two genes (*ycf3* and *clpP*) have two introns.

To investigate its taxonomic status, a total of 24 cp genome sequences of Lamiaceae species were downloaded from the NCBI database used for phylogenetic analysis. After using MAFFT V.7.149 for aligning (Katoh and Standley 2013), jModelTest v.2.1.7 (Darriba et al. 2012) was used to determine the model and the GTR + G model was the best-fitting for the cp genomes. Then Bayesian inference (BI) was performed by MrBayes v.3.2.6 (Ronquist et al. 2012) with four Solanaceae family species (NC_032724, NC_030543, MH019242, and MF818319) as outgroups. The results showed that *S. yunnanensis* was closely related to *S. miltiorrhiza* (Figure 1). Meanwhile, the present study afforded scientific evidence for resource development of Radix et Rhizoma Saliviae Miltiorrhizae and would be beneficial to taxonomy and phylogeny of Lamiaceae.

**Figure 1.**
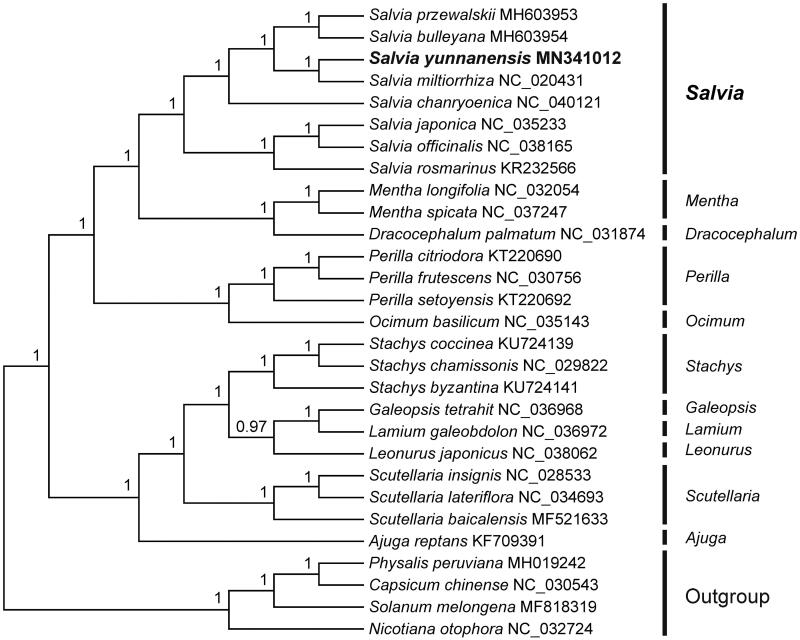
Bayesian inference (BI) tree of 25 species within the family Lamiaceae based on the complete plastome sequences using four Solanaceae family species (NC_032724, NC_030543, MH019242, and MF818319) as outgroups.
